# Phase Error Analysis and Correction for Crossed-Grating Phase-Shifting Profilometry

**DOI:** 10.3390/s21196475

**Published:** 2021-09-28

**Authors:** Fuqian Li, Wenjing Chen

**Affiliations:** Department of Opto-Electronics, Sichuan University, Chengdu 610065, China; lifuqian@stu.scu.edu.cn

**Keywords:** phase shifting profilometry, crossed grating projection, nonlinearity phase error analysis, phase error correction, inverse pattern projection

## Abstract

Crossed-grating phase-shifting profilometry (CGPSP) has great utility in three-dimensional shape measurement due to its ability to acquire horizontal and vertical phase maps in a single measurement. However, CGPSP is extremely sensitive to the non-linearity effect of a digital fringe projection system, which is not studied in depth yet. In this paper, a mathematical model is established to analyze the phase error caused by the non-linearity effect. Subsequently, two methods used to eliminate the non-linearity error are discussed in detail. To be specific, a double five-step algorithm based on the mathematical model is proposed to passively suppress the second non-linearity. Furthermore, a precoding gamma correction method based on probability distribution function is introduced to actively attenuate the non-linearity of the captured crossed fringe. The comparison results show that the active gamma correction method requires less fringe patterns and can more effectively reduce the non-linearity error compared with the passive method. Finally, employing CGPSP with gamma correction, a faster and reliable inverse pattern projection is realized with less fringe patterns.

## 1. Introduction

Phase shifting profilometry (PSP), with non-contact, full-field, high-resolution and high-precision advantages, is a popular three-dimensional (3D) shape measurement technique based on fringe projection [1,2,3]. PSP has found extensive applications in several fields such as industrial surface inspection, reverse engineering, and biomedical engineering [4,5,6]. In PSP, the height information of a tested object is encoded within the phase of the projected fringe patterns. The phase retrieved from the captured deformed fringe patterns by phase shifting algorithm is wrapped within the range from *−π* to *π* [7]. A phase unwrapping algorithm must be conducted to obtain a continuous phase map. Conventional phase unwrapping algorithms can be divided into two principal categories: spatial phase unwrapping [8,9,10] and temporal phase unwrapping [11,12,13]. Spatial phase unwrapping implemented on a single wrapped phase map is normally dependent on the unwrapping path. Due to the phase continuity assumption, it cannot handle surfaces with large discontinuities or separations. Temporal phase unwrapping can solve the phase ambiguity problem by using multi-wrapped phase maps, in which phase unwrapping is performed at each pixel independently.

In some applications of PSP, such as phase measurement deflectometry [14], inverse pattern projection (IPP) [15] and calibration of structured light measurement systems [16], it is desired that both horizontal and vertical phase maps of the tested object are recovered. Traditionally, in single carrier-frequency grating phase shifting profilometry (SGPSP), horizontal and vertical sinusoidal fringe patterns are separately projected on the tested object, and the deformed fringe patterns are recorded to recover the two orthogonal phases. To improve the measurement efficiency, Liu et al. [17] employed crossed-grating phase-shifting profilometry (CGPSP) to obtain the two orthogonal phases. Theoretically, in CGPSP, only five fringe patterns are required to obtain the two orthogonal phases.

However, CGPSP is extremely susceptible to the non-linear response of the digital fringe projection system. The non-linearity effect of the industrial camera can be neglected compared with that of the digital projector [1]. Therefore, the non-linearity effect of the projector, as the main error resource of the system [18,19], has been studied by many researchers. Some phase correction methods used in sinusoidal grating phase shifting profilometry and binary grating phase shifting profilometry (BGPSP) have been proposed. They are roughly divided into two categories: active correction and passive correction [20]. In the first category, the ideal sinusoidal fringe patterns are desired to be projected on the object. One solution is to find the most proper gamma value, which is pre-encoded in the projected fringe patterns to guarantee the captured fringes perfectly. For instance, Hoang et al. [21] used the least-square approach to calculate the system gamma value from the difference between the real phase value of the three-step phase-shifting algorithm and the true phase value obtained by a large-step phase-shifting method. Employing the statistical analysis, Guo et al. [22] calculated the gamma value from the normalized cumulative histogram of the captured fringe patterns with different backgrounds and modulations. Yu et al. [23] calculated the gamma value based on probability distribution function (PDF) of the retrieved wrapped phase. Another way to generate ideal fringes is the defocusing technique which acts as a low-pass filter. For instance, Baker et al. [24] derived the mathematical model of the defocusing technique. Lei et al. [25] generated ideal sinusoidal fringe patterns by defocusing binary fringe patterns. In the second category, the non-linearity error is passively corrected by a post-processing instead of projecting pro-encoded fringes. Pan et al. [19] proposed a simple iterative algorithm to reduce the phase error owing to non-sinusoidal waveforms. Zhang [7,26] established a look-up-table between the phase error and the corresponding phase value to compensate the phase error directly. Huang et al. [27] proposed a double three-step algorithm to obtain two distorted phases with opposite distortion direction and identical distortion amplitude, and averaged them to eliminate the distortion. The similar model based on the Hilbert transform was also proposed to eliminate the phase error [28]. Zheng et al. applied the above two methods to the phase error correction of BGPSP with projector defocusing [29]. In addition, a large-step phase-shifting method can be used to eliminate the non-linearity error [30]. However, methods with less fringe patterns and higher accuracy are the common goal. In CGPSP, fewer fringe patterns are required to obtain two orthogonal phases, but an elemental theoretical analysis of the non-linearity error and practical methods to eliminate the error are still not extensively studied.

To improve the accuracy and flexibility of CGPSP, a mathematical model to analyze the non-linearity error and practical methods to eliminate the phase error are studied in detail in this paper. Based on a polynomial model of the non-linear effect, the mathematical model is derived. To eliminate the non-linearity error, we study passive and active non-linear phase error correction methods, including a passive double five-step algorithm and an active gamma correction method based on PDF. The phase correction performance of the two methods is compared. Finally, a faster IPP is realized by employing CGPSP with gamma correction.

The rest of the paper is organized as follows: Section 2 introduces the principle of CGPSP, the principle of IPP based on CGPSP and the phase error analysis of CGPSP. Section 3 illustrates the principle of the double five-step algorithm and the gamma correction method based on PDF. Section 4 presents some experimental results. Section 5 concludes the paper.

## 2. Principle

### 2.1. Principle of Crossed-Grating Phase-Shifting Profilometry

**Figure 1:**
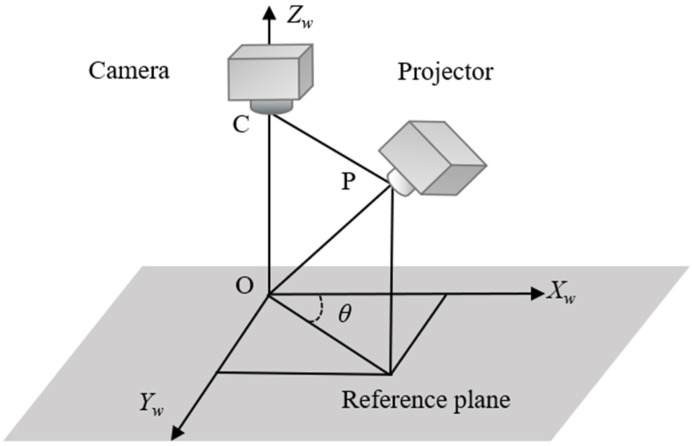
Schematic of geometric optical path for crossed-grating phase-shifting profilometry (CGPSP) measurement setup.

The schematic of geometric optical path for the CGPSP measurement setup is shown in Figure 1, where C and P are the camera photocenter and the projector photocenter respectively, O is the intersection point of the optical axis of projector and camera, and *θ* is the angle between the baseline CP and the horizontal axis *X_w_*. In order to generate the fringe deformation in both the horizontal and vertical directions, the baseline CP should have components in the horizontal and vertical directions (for example, *θ* is 45 degrees).

In the ideal measurement system, the deformed fringe patterns captured by the camera can be described as:

(1) $$I_{n}\left(x,y\right)=A\left(x,y\right)+B_{1}\left(x,y\right)\cos\left[\varphi_{x}\left(x,y\right)+\delta_{n}\right]+B_{2}\left(x,y\right)\cos\left[\varphi_{y}\left(x,y\right)+k\delta_{n}\right],$$

where *n* = 1, 2, …, *N* and *N* is the total number of phase shifts, (*x*, *y*) is the coordinate of an arbitrary point in the image, *A*(*x*, *y*) is the background intensity, *B*_1_(*x*, *y*) and *φ_x_*(*x*, *y*) are the intensity modulation and the desired phase in the horizontal direction, respectively, *B*_2_(*x*, *y*) and *φ_y_*(*x*, *y*) are the intensity modulation and the desired phase in the vertical direction respectively, *δ_n_* = 2*πn/N* is the phase-shifting amount, *k* is an integer within the range of [2, *N*/2)∪(*N*/2, *N* − 2] and controls the phase-shifting amount in the vertical direction. Because there are five unknown variables in Equation (1), at least five images should be used to extract the phase information. For convenience, (*x*, *y*) is omitted in some complex expressions hereafter.

The measured phases in the horizontal and vertical direction can be extracted by the least-squares algorithm:

(2) $$\varphi_{xw}=-\arctan\left(\frac{\sum_{n=1}^{N}I_{n}\sin\left(\delta_{n}\right)}{\sum_{n=1}^{N}I_{n}\cos\left(\delta_{n}\right)}\right),\varphi_{yw}=-\arctan\left(\frac{\sum_{n=1}^{N}I_{n}\sin\left(k\delta_{n}\right)}{\sum_{n=1}^{N}I_{n}\cos\left(k\delta_{n}\right)}\right).$$

The phases *φ_xw_* and *φ_yw_* are wrapped within the range of [−π, π). To recover unambiguous phases even in the presence of large discontinuities or isolated objects, simple and robust three-frequency temporal phase unwrapping (3FTPU) is carried out. For the horizontal phase unwrapping, the 3FTPU algorithm can be simply described as:

(3) $$\{\begin{array}{c} \varphi_{xu1}=\varphi_{xw1}\\ \varphi_{xul}=\varphi_{xwl}+2\pi \cdot Round\left[\frac{\left(f_{l}/1\right)\varphi_{xu1}-\varphi_{xwl}}{2\pi }\right]\\ \varphi_{xuh}=\varphi_{xwh}+2\pi \cdot Round\left[\frac{\left(f_{h}/f_{l}\right)\varphi_{xul}-\varphi_{xwh}}{2\pi }\right] \end{array},$$

where 1, *f_l_* and *f_h_* (commonly *f_h_* = *f_l_*^2^) are the carrier frequencies of three sets of crossed fringes, and subscripts l and h denote ‘low frequency’ and ‘high frequency’, respectively. *φ_xw_*_1_, *φ_xwl_* and *φ_xwh_* denote the unwrapped phases of the single-, low- and high-frequency fringes, respectively. *Round*[∙] is the operation to obtain the closest integer value.

In 3FTPU based on CGPSP, three set of fringes with frequencies of 1, *f_l_* and *f_h_* in the horizontal and vertical directions are required. Theoretically, two orthogonal phase maps can be extracted from 15 captured fringe patterns. As a comparison, in traditional 3FTPU based on SGPSP [12], 18 fringe patterns are required, employing the three-step phase-shifting method. Twenty-four fringe patterns are required, employing the four-step phase-shifting method. Therefore, CGPSP normally requires fewer fringes to obtain two orthogonal phase maps compared with SGPSP.

### 2.2. Principle of Crossed-Grating Phase-Shifting Profilometry

**Figure 2:**
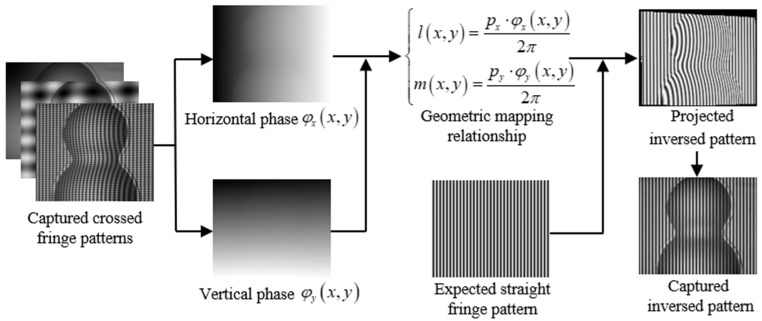
Schematic diagram of inverse pattern projection (IPP) based on CGPSP.

IPP has been widely used in many fields such as industrial surface detection [31], and augmented reality [32]. In traditional IPP, horizontal and vertical phase-shifting fringe patterns are separately projected to obtain horizontal and vertical absolute phases which are used to establish the geometric mapping relationship between the projector pixel and the camera pixel. If the CGPSP method is used, the mapping relationship can be established with fewer fringes. Taking the generation of an inverse straight fringe pattern as an example, the flow chart as shown in Figure 2 illustrates the whole process of IPP based on CGPSP. For simplicity, the first frames of three sets of captured crossed fringe patterns are shown in Figure 2. Firstly, the horizontal and vertical absolute phases of a target object are recovered by CGPSP based on 3FTPU. With the two phases, the geometric mapping relationship between the projector pixel (*l*, *m*) and the camera pixel (*x*, *y*) can be established:

(4) $$l\left(x,y\right)=\frac{p_{x}\cdot \varphi_{x}\left(x,y\right)}{2\pi },m\left(x,y\right)=\frac{p_{y}\cdot \varphi_{y}\left(x,y\right)}{2\pi },$$

where *φ_x_*(*x*, *y*) and *φ_y_*(*x*, *y*) denote the horizontal and vertical phases respectively, *p_x_* and *p_y_* are the crossed fringe pitches in the horizontal and vertical directions respectively. The high accuracy of the two orthogonal phases guarantees the precision of the geometric mapping relationship in Equation (4). Based on the geometric mapping relationship and an expected pattern (straight fringe), a projected inverse pattern (deformed fringe) is generated. In the same projector-camera measurement system, after projecting the inverse pattern on the object, a non-distorted fringe pattern on the surface of the object can be collected by the camera.

### 2.3. Phase Error Analysis for Crossed-Grating Phase-Shifting Profilometry

**Figure 3:**
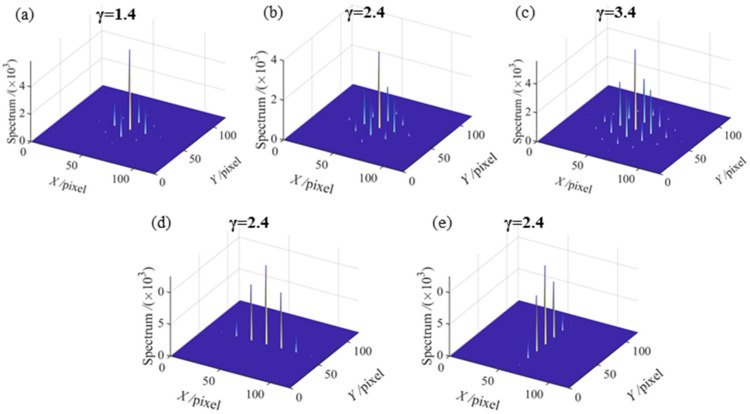
Frequency spectra of three simulated fringes with gamma distortion: (**a**–**c**) frequency spectra of crossed fringe with gamma value 1.4, 2.4 and 3.4, respectively; (**d**) frequency spectrum of vertical sinusoidal fringe with gamma value 2.4; (**e**) frequency spectrum of horizontal sinusoidal fringe with gamma value 2.4.

In practice, considering the non-linearity effect of the measurement system, the actual captured fringe patterns *I_n_*′ can be theoretically described as the gamma power of the ideal captured fringe patterns *I_n_* [33].

(5) $$I_{n}^{\prime }=I_{n}^{\gamma },$$

where *γ* is the gamma value of the system and can be a non-integer value. To facilitate the analysis of the non-linear error, Equation (5) can be approximated by a polynomial function [34]:

(6) $$I_{n}^{\prime }=\varepsilon_{0}+\varepsilon_{1}I_{n}+\varepsilon_{2}{\left(I_{n}\right)}^{2}+\varepsilon_{3}{\left(I_{n}\right)}^{3}+\ldots ,$$

where *ε*_0_, *ε*_1_, *ε*_2_, *ε*_3_ are coefficients. Substituting Equation (1) into Equation (6), the actual captured crossed fringe patterns can be calculated as

(7) $$\begin{array}{l} I_{n}^{\prime }=a_{0}+\sum_{i=1}^{\infty }a_{i}\cos\left[i\left(\varphi_{x}+\delta_{n}\right)\right]+\sum_{i=1}^{\infty }b_{i}\cos\left[i\left(\varphi_{y}+k\delta_{n}\right)\right]+\\ \sum_{i=2}^{\infty }\sum_{j=1}^{i-1}e_{ij}\cos\left[j\left(\varphi_{x}+\delta_{n}\right)\right]\cos\left[\left(i-j\right)\left(\varphi_{y}+k\delta_{n}\right)\right], \end{array}$$

where *a*_0_ is the background intensity, *a_i_*, *b_j_* and *e_ij_* denote the amplitudes of horizontal and vertical harmonic components and crosstalk components, respectively. It is clear that the phase error will be introduced by horizontal and vertical high-order harmonics and crosstalk components. It has been verified that the harmonic amplitude decreases rapidly with the increasing of its frequency in SGPSP [19]. Non-linear system response makes the harmonic amplitude distribution of the captured sinusoidal fringe follow the conclusion regardless of the carrier frequency in the horizontal direction or vertical direction. Therefore, the conclusion is also valid in CGPSP. A simulation is added to confirm the conclusion in CGPSP more clearly. The spectra of the simulated crossed fringe with different gamma values 1.4, 2.4, and 3.4, as shown in Figure 3a–c respectively, illustrate the conclusion. As a comparison, the frequency spectra of a simulated vertical sinusoidal fringe and a simulated horizontal sinusoidal fringe distorted by gamma value 2.4 are shown in Figure 3d,e, respectively. Compared with the single carrier-frequency fringes, the crossed fringe has more complex spectra distribution.

When the parameter *k* is set to two in Equation (1), a full-cycle phase shifting within 4*π* is performed in the vertical direction. Setting *k* to 2 and substituting Equation (7) into Equation (2), we derive two wrapped phase expressions which are related to the total number of phase shifts *N* (*N* ≥ 5).

1. When *N* is an odd number, the wrapped phases *φ_xw_*′ and *φ_yw_*′ are derived as:
  (8) $$\begin{array}{l} \varphi_{xw}^{\prime }=\arctan\{\frac{a_{1}\sin\varphi_{x}-\sum_{m=1}^{\infty }a_{mN-1}\sin\left[\left(mN-1\right)\varphi_{x}\right]+\sum_{m=1}^{\infty }a_{mN+1}\sin\left[\left(mN+1\right)\varphi_{x}\right]-\sum_{m=1}^{\infty }b_{\frac{\left(2m-1\right)N-1}{2}}\sin\left[\frac{\left(2m-1\right)N-1}{2}\varphi_{y}\right]}{a_{1}\cos\varphi_{x}+\sum_{m=1}^{\infty }a_{mN-1}\cos\left[\left(mN-1\right)\varphi_{x}\right]+\sum_{m=1}^{\infty }a_{mN+1}\cos\left[\left(mN+1\right)\varphi_{x}\right]+\sum_{m=1}^{\infty }b_{\frac{\left(2m-1\right)N-1}{2}}\cos\left[\frac{\left(2m-1\right)N-1}{2}\varphi_{y}\right]}\\ \frac{+\sum_{m=1}^{\infty }b_{\frac{\left(2m-1\right)N+1}{2}}\sin\left[\frac{\left(2m-1\right)N+1}{2}\varphi_{y}\right]-\frac{e_{21}}{2}\sin\left(\varphi_{x}-\varphi_{y}\right)+F\left(\varphi_{x},\varphi_{y}\right)}{+\sum_{m=1}^{\infty }b_{\frac{\left(2m-1\right)N+1}{2}}\cos\left[\frac{\left(2m-1\right)N+1}{2}\varphi_{y}\right]+\frac{e_{21}}{2}\cos\left(\varphi_{x}-\varphi_{y}\right)+G\left(\varphi_{x},\varphi_{y}\right)}\}, \end{array}$$
  (9) $$\begin{array}{l} \varphi_{yw}^{\prime }=\arctan\{\frac{b_{1}\sin\varphi_{y}-\sum_{m=1}^{\infty }b_{mN-1}\sin\left[\left(mN-1\right)\varphi_{y}\right]+\sum_{m=1}^{\infty }b_{mN+1}\sin\left[\left(mN+1\right)\varphi_{y}\right]+a_{2}\sin\left(2\varphi_{x}\right)-\sum_{m=1}^{\infty }a_{mN-2}\sin\left[\left(mN-2\right)\varphi_{x}\right]}{b_{1}\cos\varphi_{y}+\sum_{m=1}^{\infty }b_{mN-1}\cos\left[(mN-1)\varphi_{y}\right]+\sum_{m=1}^{\infty }b_{mN+1}\cos\left[\left(mN+1\right)\varphi_{y}\right]+a_{2}\cos\left(2\varphi_{x}\right)+\sum_{m=1}^{\infty }a_{mN-2}\cos\left[\left(mN-2\right)\varphi_{x}\right]}\\ \frac{+\sum_{m=1}^{\infty }a_{mN+2}\sin\left[\left(mN+2\right)\varphi_{x}\right]+H\left(\varphi_{x},\varphi_{y}\right)}{+\sum_{m=1}^{\infty }a_{mN+2}\cos\left[\left(mN+2\right)\varphi_{x}\right]+I\left(\varphi_{x},\varphi_{y}\right)}\}, \end{array}$$
  where *m* is a positive integer for describing the harmonic order. The functions *F*(*φ_x_*, *φ_y_*) and *G*(*φ_x_*, *φ_y_*) in Equation (8), and *H*(*φ_x_*, *φ_y_*) and *I*(*φ_x_*, *φ_y_*) in Equation (9) are regarded as the high-order crossed phase error. They are the summation of periodic sinusoidal and cosinusoidal harmonic functions with variables *φ_x_* and *φ_y_*, but their mathematical expressions are too complex to be written as explicit functions. Their magnitudes decrease with the increasing of the total number of phase shifts *N*. Seen from Equations (8) and (9), both *φ_xw_*′ and *φ_yw_*′ include periodic phase error caused by the horizontal and vertical high-order harmonics and the crosstalk components. Equation (8) shows that the horizontal phase error is introduced by the *mN* ± 1th horizontal harmonics, the [(2*m* − 1)*N* − 1]/2th vertical harmonics and the crossed phases −[*e*_21_sin(*φ_x_* − *φ_y_*)]/2 + *F*(*φ_x_*, *φ_y_*) and [*e*_21_cos(*φ_x_* − *φ_y_*)]/2 + *G*(*φ_x_*, *φ_y_*). Equation (9) shows that the vertical phase error is induced by the 2nd and *mN* ± 2th horizontal harmonics, the *mN* ± 1th vertical harmonics and the crossed phases *H*(*φ_x_*, *φ_y_*) and *I*(*φ_x_*, *φ_y_*). For instance, when *N* = 5 and *m* = 1, 2, *φ_xw_*′ includes the horizontal phase error introduced by the horizontal harmonics 4, 6, 9, 11, vertical harmonics 2, 3, 7, 8, and the crossed phases −[*e*_21_sin(*φ_x_* − *φ_y_*)]/2 + *F*(*φ_x_*, *φ_y_*)|*_N_*_=5_ and [*e*_21_cos(*φ_x_* − *φ_y_*)]/2 + *G*(*φ_x_*, *φ_y_*)|*_N_*_=5_. *φ_yw_*′ includes the vertical phase error introduced by the horizontal harmonics 2, 3, 7, 8, 12, the vertical harmonics 4, 6, 9, 11, and the crossed phases *H*(*φ_x_*, *φ_y_*)|*_N_*_=5_ and *I*(*φ_x_*, *φ_y_*)|*_N_*_=5_.
2. When *N* is an even number, *φ_xw_*′ and *φ_yw_*′ are derived as:
  (10) $$\varphi_{xw}^{\prime }=\arctan\left\{\frac{a_{1}\sin\varphi_{x}-\sum_{m=1}^{\infty }a_{mN-1}\sin\left[\left(mN-1\right)\varphi_{x}\right]+\sum_{m=1}^{\infty }a_{mN+1}\sin\left[\left(mN+1\right)\varphi_{x}\right]-\frac{e_{21}}{2}\sin\left(\varphi_{x}-\varphi_{y}\right)+J\left(\varphi_{x},\varphi_{y}\right)}{a_{1}\cos\varphi_{x}+\sum_{m=1}^{\infty }a_{mN-1}\cos\left[(mN-1)\varphi_{x}\right]+\sum_{m=1}^{\infty }a_{mN+1}\cos\left[\left(mN+1\right)\varphi_{x}\right]+\frac{e_{21}}{2}\cos\left(\varphi_{x}-\varphi_{y}\right)+K\left(\varphi_{x},\varphi_{y}\right)}\right\},$$
  (11) $$\begin{array}{l} \varphi_{yw}^{\prime }=\arctan\{\frac{b_{1}\sin\varphi_{y}-\sum_{m=1}^{\infty }b_{\frac{mN}{2}-1}\sin\left[\left(\frac{mN}{2}-1\right)\varphi_{y}\right]+\sum_{m=1}^{\infty }b_{\frac{mN}{2}+1}\sin\left[\left(\frac{mN}{2}+1\right)\varphi_{y}\right]+a_{2}\sin\left(2\varphi_{x}\right)-\sum_{m=1}^{\infty }a_{mN-2}\sin\left[\left(mN-2\right)\varphi_{x}\right]}{b_{1}\cos\varphi_{y}+\sum_{m=1}^{\infty }b_{\frac{mN}{2}-1}\cos\left[\left(\frac{mN}{2}-1\right)\varphi_{y}\right]+\sum_{m=1}^{\infty }b_{\frac{mN}{2}+1}\cos\left[\left(\frac{mN}{2}+1\right)\varphi_{y}\right]+a_{2}\cos\left(2\varphi_{x}\right)+\sum_{m=1}^{\infty }a_{mN-2}\cos\left[(mN-2)\varphi_{x}\right]}\\ \frac{+\sum_{m=1}^{\infty }a_{mN+2}\sin\left[\left(mN+2\right)\varphi_{x}\right]+L\left(\varphi_{x},\varphi_{y}\right)}{+\sum_{m=1}^{\infty }a_{mN+2}\cos\left[\left(mN+2\right)\varphi_{x}\right]+M\left(\varphi_{x},\varphi_{y}\right)}\}, \end{array}$$

The four functions *J*(*φ_x_*, *φ_y_*), *K*(*φ_x_*, *φ_y_*), *L*(*φ_x_*, *φ_y_*) and *M*(*φ_x_*, *φ_y_*) are also composed of the summation of periodic sinusoidal and cosinusoidal harmonic functions with variables *φ_x_* and *φ_y_*. Their magnitudes decrease with the increasing of the total number of phase shifts *N*. Seen from Equations (10) and (11), the phase expressions are different with those in the odd condition. However, periodic phase error caused by horizontal and vertical high-order harmonics and crosstalk components still exists in the calculated phases. Equations (10) and (11) reveal that the horizontal phase error is induced by the *mN* ± 1th horizontal harmonics and the crossed phases −[*e*_21_sin(*φ_x_* − *φ_y_*)]/2 + *J*(*φ_x_*, *φ_y_*) and [*e*_21_cos(*φ_x_* − *φ_y_*)]/2 + *K*(*φ_x_*, *φ_y_*), and the vertical phase error is induced by the 2nd and mN ± 2th horizontal harmonics, the *mN*/2 ± 1th vertical harmonics and the crossed phases *L*(*φ_x_*, *φ_y_*) and *M*(*φ_x_*, *φ_y_*).

For clarity, a summary is given as follows:

- There is always the crossed-phase component *φ_x_* − *φ_y_* in the horizontal distorted phase;
- There is always the phase error induced by the 2nd horizontal harmonic in the vertical distorted phase;
- The crossed phase component *φ_x_* − *φ_y_* and the 2nd horizontal harmonic in Equations (8)–(11) make the large-step phase-shifting method invalid to eliminate the non-linear error in CGPSP.

When parameter *k* is set to other integer within the range of [2, *N*/2)∪(*N*/2, *N* − 2], similar phase distribution can be derived, which can be summarized as below:

- When *N* ≥ 2*k* + 1, there is always the crossed phase component (*k* − 1)*φ_x_* − *φ_y_* in the horizontal distorted phase;
- When *N* ≥ 2*k* + 1, there is always the phase error induced by the *k*th horizontal harmonic in the vertical distorted phase.

## 3. Phase Error Correction for Crossed-Grating Phase-Shifting Profilometry

The above analysis results show that the non-linearity effect in CGPSP is quite serious and complicated. Therefore, it is necessary to remove the non-linear error for the further application of CGPSP. In this section, a double five-step algorithm is presented to passively eliminate the second non-linearity. In addition, an attractive gamma correction method based on PDF [23] is discussed in detail as well.

### 3.1. Double Five-Step Algorithm

Referring to the double three-step algorithm [27], the double five-step algorithm is proposed to eliminate the second non-linearity effect in CGPSP which is the main component of the non-linearity effect normally. Considering the non-linearity up to the second order and setting *N* to 5, Equations (8) and (9) can be simplified and the corresponding unwrapped phases can be expressed as:

(12) $$\{\begin{array}{c} \varphi_{xu}^{\prime }=U\left\{\arctan\left[\frac{a_{1}\sin\varphi_{x}-b_{2}\sin2\varphi_{y}-\frac{e_{21}}{2}\sin\left(\varphi_{x}-\varphi_{y}\right)}{a_{1}\cos\varphi_{x}+b_{2}\cos2\varphi_{y}+\frac{e_{21}}{2}\cos\left(\varphi_{x}-\varphi_{y}\right)}\right]\right\}\\ \varphi_{yu}^{\prime }=U\left\{\arctan\left[\frac{b_{1}\sin\varphi_{y}+a_{2}\sin2\varphi_{x}-\frac{e_{21}}{2}\sin\left(\varphi_{x}+\varphi_{y}\right)}{b_{1}\cos\varphi_{y}+a_{2}\cos2\varphi_{x}+\frac{e_{21}}{2}\cos\left(\varphi_{x}+\varphi_{y}\right)}\right]\right\} \end{array},$$

where *U*{∙} is the operation to unwrap the wrapped phase. The phase error in the two directions can be derived as follows:

(13) $$\{\begin{array}{c} \Delta \varphi_{xu}^{\prime }=\varphi_{xu}^{\prime }-\varphi_{x}=U\left\{\arctan\left[\frac{-b_{2}\sin\left(2\varphi_{y}+\varphi_{x}\right)-\frac{e_{21}}{2}\sin\left(2\varphi_{x}-\varphi_{y}\right)}{a_{1}+b_{2}\cos\left(2\varphi_{y}+\varphi_{x}\right)+\frac{e_{21}}{2}\cos\left(2\varphi_{x}-\varphi_{y}\right)}\right]\right\}\\ \Delta \varphi_{yu}^{\prime }=\varphi_{yu}^{\prime }-\varphi_{y}=U\left\{\arctan\left[\frac{a_{2}\sin\left(2\varphi_{x}-\varphi_{y}\right)-\frac{e_{21}}{2}\sin\left(2\varphi_{y}+\varphi_{x}\right)}{b_{1}+a_{2}\cos\left(2\varphi_{x}-\varphi_{y}\right)+\frac{e_{21}}{2}\cos\left(2\varphi_{y}+\varphi_{x}\right)}\right]\right\} \end{array},$$

where *φ_x_* and *φ_y_* are the ideal phases. In practice, *a*_1_ is much larger than *b*_2_ and *e*_21_/2, and *b*_1_ is much larger than *a*_2_ and *e*_21_/2. Equation (13) can be simplified as:

(14) $$\{\begin{array}{c} \Delta \varphi_{xu}^{\prime }\approx U\left\{\arctan\left[\frac{-b_{2}\sin(2\varphi_{y}+\varphi_{x})-\frac{e_{21}}{2}\sin(2\varphi_{x}-\varphi_{y})}{a_{1}}\right]\right\}\\ \Delta \varphi_{yu}^{\prime }\approx U\left\{\arctan\left[\frac{a_{2}\sin\left(2\varphi_{x}-\varphi_{y}\right)-\frac{e_{21}}{2}\sin\left(2\varphi_{y}+\varphi_{x}\right)}{b_{1}}\right]\right\} \end{array}.$$

If an extra phase offset of *π* is introduced into the ideal fringes, two extra measured phases *φ_xu_*″ and *φ_yu_*″ can be obtained and the corresponding phase errors can be derived as:

(15) $$\{\begin{array}{c} \Delta \varphi_{xu}^{\prime \prime }\approx -U\left\{\arctan\left[\frac{-b_{2}\sin(2\varphi_{y}+\varphi_{x})-\frac{e_{21}}{2}\sin(2\varphi_{x}-\varphi_{y})}{a_{1}}\right]\right\}\\ \Delta \varphi_{yu}^{\prime \prime }\approx -U\left\{\arctan\left[\frac{a_{2}\sin\left(2\varphi_{x}-\varphi_{y}\right)-\frac{e_{21}}{2}\sin\left(2\varphi_{y}+\varphi_{x}\right)}{b_{1}}\right]\right\} \end{array}.$$

It is obvious that ∆*φ_xu_*′ *=* −∆*φ_xu_*″ and ∆*φ_yu_*′ *=* −∆*φ_yu_*″. Therefore, the second non-linearity can be eliminated by averaging the two sets of measured phases:

(16) $$\varphi_{x}=\frac{\varphi_{xu}^{\prime }+\varphi_{xu}^{\prime \prime }-\pi }{2},\varphi_{y}=\frac{\varphi_{yu}^{\prime }+\varphi_{yu}^{\prime \prime }-\pi }{2}.$$

**Figure 4:**
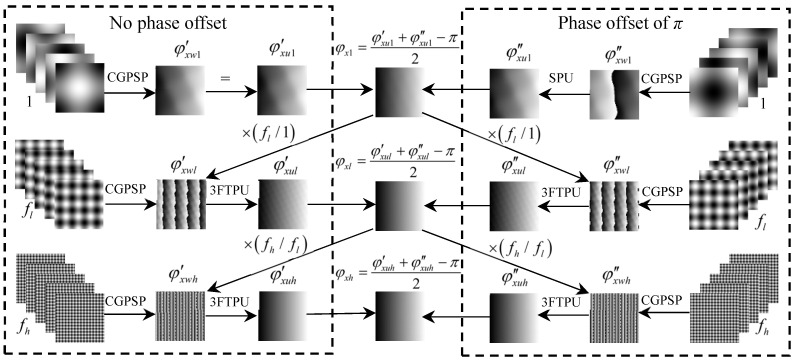
Combination flowchart of the double five-step algorithm and the three-frequency temporal phase unwrapping (3FTPU) algorithm.

To clearly explain the process of retrieving the absolute phases of 3FTPU based on double five-step algorithm, Figure 4 shows the flowchart of calculating the horizontal absolute phase, in which 1, *f_l_* and *f_h_* are the carrier frequencies of three sets of crossed fringes, respectively. Subscripts *w* and *u* denote ‘wrapped phase’ and ‘unwrapped phase’, respectively. SPU denote the spatial phase unwrapping operation. Three steps are used to describe the procedure of calculating the horizontal phase:

- Step 1: Two unwrapped phases *φ_xw_*_1_′ and *φ_xw_*_1_″ with non-linearity error are obtained from the single-frequency crossed fringes with and without the initial phase offset of *π*, respectively. Then, an unwrapped phase *φ_x_*_1_ eliminated the second non-linearity error obtained by the double five-step algorithm.
- Step 2: Two wrapped phases *φ_xwl_*′ and *φ_xwl_*″ are firstly obtained from the low-frequency (*f_l_*) crossed fringes with and without the initial phase offset of *π*, respectively. Then, combining the unwrapped phase *φ_x_*_1_ obtained in Step 1, the two wrapped phases are unwrapped to obtain *φ_xul_*′ and *φ_xul_*″, respectively. Finally, an unwrapped phase *φ_xl_* without the second non-linearity error can be obtained by the double five-step algorithm.
- Step 3: Similarly, two wrapped phases *φ_xwh_*′ and *φ_xwh_*″ respectively obtained from high-frequency (*f_h_*) crossed fringes with and without the initial phase offset of *π* are unwrapped to obtain *φ_xuh_*′ and *φ_xuh_*″, employing the unwrapped phase *φ_xl_* obtained in Step 2. The final corrected phase *φ_xh_* can be obtained by the double five-step algorithm.

A similar procedure can be performed to obtain the vertical phase from the crossed fringes.

**Figure 5:**
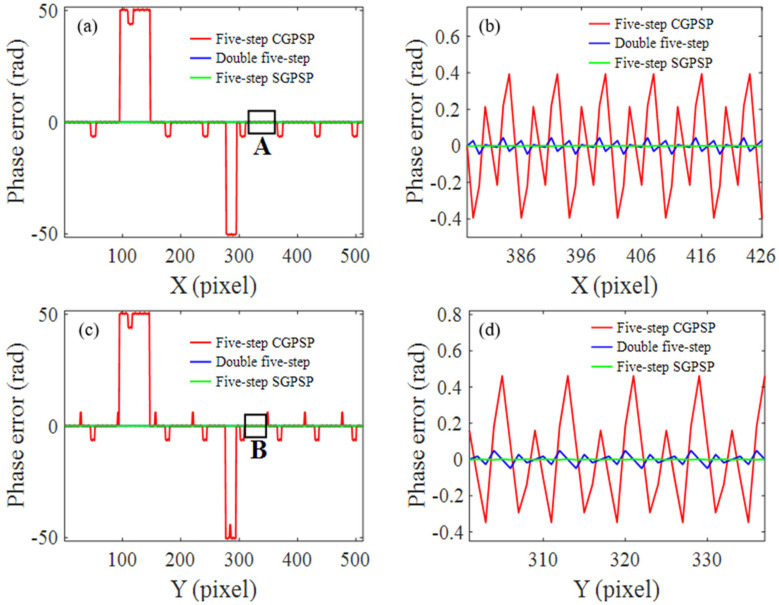
Phase error distribution of five-step CGPSP, the double five-step algorithm and five-step SGPSP: (**a**) the horizontal phase error; (**b**) zoomed view of area A in (**a**); (**c**) the vertical phase error; (**d**) zoomed view of area B in (**c**).

A simulation is conducted to investigate the performance of the double five-step algorithm. The size of the simulated fringe patterns is 512 × 512 pixels. Three sets of fringes with the number of fringe periods of 1, 8 and 64 are used in 3FTPU. The gamma value of the system is set as 2.4, which leads to obvious 2nd and 3rd non-linearity. The simulated phase error of the double five-step algorithm, the five-step CGPSP method and the traditional five-step SGPSP method are compared. The 400th row of the horizontal phase errors and the 114th column of the vertical phase errors are shown in Figure 5a,c, respectively. For clarity, Figure 5b,d show the zoomed results of area A in Figure 5a and area B in Figure 5c, respectively. The STD values of the phase errors and the number of fringe patterns are given in Table 1. As shown in Figure 5a,c, abnormal phase jump errors emerge in five-step CGPSP because the non-linearity errors in the wrapped phases lead to fringe order errors in phase unwrapping. The double five-step algorithm effectively improves the phase accuracy by eliminating the second non-linearity. The five-step SGPSP performs better than the double five-step algorithm because it eliminates the second and third non-linearity. Anyway, the double five-step CGPSP provides a convenient way to eliminate second non-linearity in the measurement system where the physical crossed grating is designed to produce the structured fringe. If only the second non-linearity is included in the crossed fringe patterns, the accuracy of five-step CGPSP is as same as that of five-step SGPSP.

### 3.2. Gamma Correction Method Based on Probability Distribution Function

The non-linearity error in CGPSP is so complicated that it is difficult to passively eliminate all the high-order harmonics and the crosstalk components. An active correction method, the gamma correction method based on PDF [23], is used to eliminate the phase error in CGPSP.

Theoretically, if an appropriate value *γ_p_* which nearly equals 1/*γ* is found to preprocess the projected fringes, the non-linearity of the system can be eliminated. The captured crossed fringes can be rewritten as:

(17) $$I_{n}^{\prime }=I_{n}^{\gamma /\gamma_{p}}.$$

The gamma correction method based on PDF [23] can be used to calculate the pre-encoded value *γ_p_*. In practice, considering the defocusing effect of the digital projector with a large aperture, Equation (17) is rewritten as [23]:

(18) $$I_{n}^{\prime }=C_{1}I_{n}^{\gamma_{a}/\gamma_{p}+\gamma_{b}}+C_{2},$$

where *C*_1_, *C*_2_, *γ_a_* and *γ_b_* are system parameters. By projecting two sets of vertical sinusoidal fringe patterns with different pre-encoded gamma values, *γ_a_* and *γ_b_* can be calculated based on the PDF method. Then, setting *γ_a_*/*γ_p_* + *γ_b_* = 1, the desired pre-encoded value *γ_p_* can be obtained.

**Figure 6:**
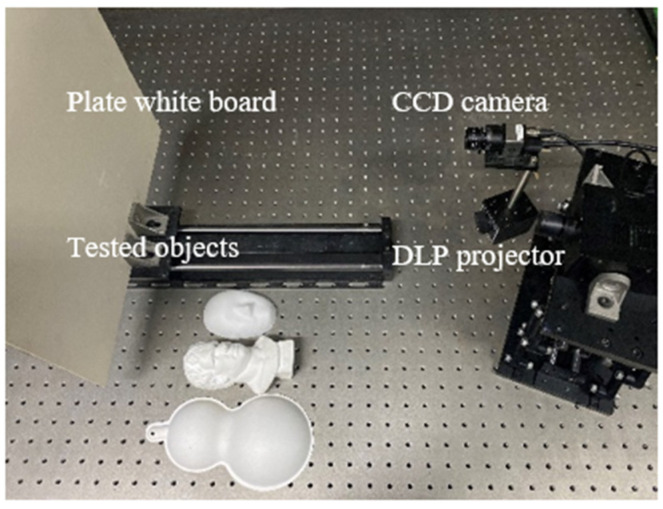
Experimental setup.

Theoretically, the pre-encoded value *γ_p_* reflects the non-linearity of the system and can be calculated from either the captured horizontal or vertical sinusoidal fringe patterns. For instance, using the experimental setup as shown in Figure 6 and projecting the horizontal and vertical sinusoidal fringe patterns, we calculated two sets of pre-encoded values in different measurement conditions (e.g., the projector and camera were adjusted slightly). The results, as shown in Table 2, indicate that the two pre-encoded values in each case are close, which coincides with the theoretical analysis.

## 4. Experiments and Discussion

The experimental setup is shown in Figure 6, including a DLP projector with the resolution of 1280 × 800 (GVD PDC03), a CCD camera with the resolution of 1280 × 1024 (IDS UI-124xSE-M), a plate white board and some tested objects. To generate fringe deformation in both horizontal and vertical directions, the baseline of the projector and the camera has components in the horizontal and vertical direction of the board. The 3FTPU algorithm is used in the experiment; 1, 8 and 64 are selected as the number of fringe period of the single-frequency, low-frequency and high-frequency crossed fringes, respectively.

### 4.1. Phase Calculation of Crossed-Grating Phase-Shifting Profilometry

**Figure 7:**
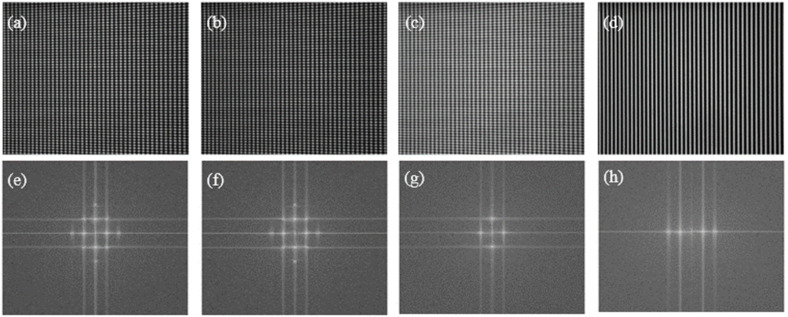
Captured fringe patterns and their frequency spectra: (**a**,**e**) crossed fringe; (**b**,**f**) crossed fringe with an initial phase offset of *π*; (**c**,**g**) crossed fringe with gamma correction; (**d**,**h**) vertical sinusoidal fringe.

First, the accuracy of the double five-step algorithm and the gamma correction method were compared by measuring the flat white board. Traditional 16-step SGPSP were used to obtain the ideal horizontal and vertical phases of the white board. The phase error was obtained by subtracting the measured phase from the ideal phase in each direction. The fringe patterns and their spectra are shown in Figure 7. Figure 7a shows a captured crossed fringe pattern. Figure 7b shows a captured crossed fringe pattern with an initial phase offset of *π*. Figure 7c is a captured crossed fringe with gamma correction and Figure 7d is a vertical sinusoidal fringe. Figure 7e–h show the corresponding frequency spectra of the above four fringe patterns. There are obvious second harmonic and crosstalk components in Figure 7e,f, while these harmonics are eliminated by the gamma correction method, as shown in Figure 7g. The captured vertical sinusoidal fringe pattern only contains obvious second harmonic besides the direct component and the fundamental component, as shown in Figure 7h.

**Figure 8:**
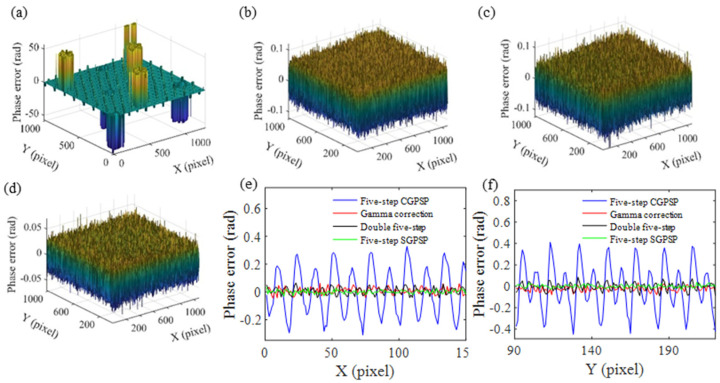
The phase error distributions of four methods: (**a**) horizontal phase error of five-step CGPSP; (**b**) horizontal phase error of the double five-step algorithm; (**c**) horizontal phase error of five-step CGPSP with gamma correction; (**d**) horizontal phase error of five-step SGPSP; (**e**) a section of the 300th row of the horizontal phase errors; (**f**) a section of the 456th column of the vertical phase errors.

Taking the horizontal phase as example, Figure 8a–d show the phase error maps obtained by five-step CGPSP, the double-five algorithm, five-step CGPSP with gamma correction and five-step SGPSP, respectively. For clarity, a section of the 300th row of the horizontal phase errors and a section of the 456th column of the vertical phase errors are shown in Figure 8e,f, respectively. Apparently, there are serious phase jump errors caused by the fringe order errors in five-step CGPSP. The double five-step algorithm apparently reduces the non-linearity error. The phase error of the gamma correction method is less than that of the double five-step algorithm, and the phase error of the five-step SGPSP is smallest. The quantitative comparison results are listed in Table 3. The results of the double five-step algorithm and the five-step SGPSP method coincide with their simulation results. The gamma correction method performs better than the double five-step algorithm. In addition, the error of the gamma correction method is bigger than that of the five-step SGPSP method, because there are more low-intensity areas in the crossed fringe pattern compared with the single carrier-frequency fringe, which decreases the signal-to-noise ratio. However, the error is acceptable and fewer fringe patterns are required in the gamma correction method.

**Figure 9:**
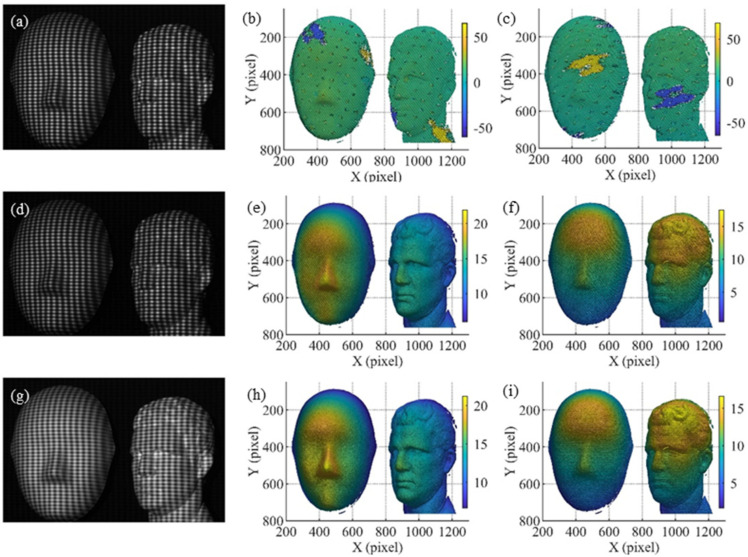
Measurement results of three methods: (**a**–**c**) results of the five-step CGPSP; (**d**–**f**) results of the double five-step algorithm; (**g**–**i**) results of the five-step CGPSP with gamma correction. (Unit of phase: radians).

In the second experiment, a couple of isolated surfaces were measured. The results are shown in Figure 9, where the first, second and third rows correspond to the measurement results of five-step CGPSP, the double five-step algorithm and five-step CGPSP with gamma correction, respectively. The captured high-frequency crossed fringe patterns, the horizontal and vertical phases of the three methods are shown in the first, second and third columns of Figure 9, respectively. The phases retrieved by five-step CGPSP are distorted seriously by the non-linearity effect, as shown in Figure 9b,c. The double five-step CGPSP effectively attenuates the non-linearity error, but there is still the residue phase error in the surfaces as shown in Figure 9e,f. Because the method only eliminates the second non-linearity. Figure 9h,i show that the gamma correction method almost removes the non-linearity error.

The experiments indicate that CGPSP with gamma correction can more effectively eliminate the non-linearity error and requires less fringes compared with the double five-step algorithm. However, the double five-step algorithm can provide convenience to eliminate the non-linear error in the case of using the physical grating to generate the crossed fringe pattern.

### 4.2. Inverse Pattern Projection Based on CGPSP

**Figure 10:**
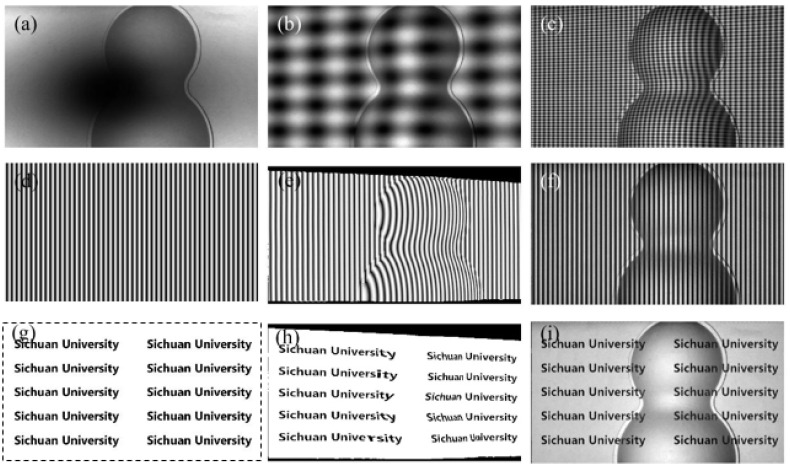
Measurement results of IPP based on CGPSP: (**a**–**c**) deformed crossed fringes with the number of fringe period of 1, 8, 64; (**d**) defined straight fringe pattern; (**e**) projected inverse fringe pattern; (**f**) captured pattern after projecting (**e**); (**g**) defined character pattern; (**h**) projected inverse character pattern; (**i**) captured pattern after projecting (**g**).

The five-step CGPSP method with gamma correction is applied in the IPP technique. A gourd model shown in Figure 10a was measured. Three captured crossed fringe patterns with the number of fringe periods of 1, 8 and 64 are shown in Figure 10a–c, respectively. We defined an expected straight fringe pattern and a character pattern which could be ‘seen’ by the CCD camera, as shown in Figure 10d,g. Two corresponding inverse patterns were generated by the IPP technique, as shown in Figure 10e,h. The resulting images captured by the CCD camera are shown in Figure 10f,i. The results verify that the geometric mapping relationship between the projector pixel and the camera pixel are correctly established by IPP based on CGPSP with fewer fringes.

**Figure 11:**
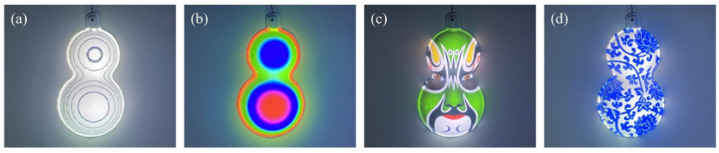
Three-dimensional display results of four encoding methods. (**a**) 3D display of the contour encoding. (**b**) 3D display of the color encoding. (**c**,**d**) 3D display of the designed color pattern encoding.

An application of IPP based on CGPSP with gamma correction was carried out to show the encoded two-dimensional (2D) pattern on the 3D object, as an augmented reality display. A 3LCD projector (Epson CB-X29) was used to project the crossed fringe patterns and the inverse patterns. The height distribution of the gourd model was firstly obtained employing the phase-to-height mapping technique [35]. Then, with the height information and the geometric mapping relationship established in advance, a contour-line inverse pattern and a color-coding inverse pattern were generated. Finally, the inverse patterns were projected on the gourd model. The resulting images captured by a camera are shown in Figure 11a,b, respectively. Another two inverse patterns of the designed color images were also generated and projected on the object. The captured images are shown in Figure 11c,d. These encoded 2D patterns fit well on the 3D model, which enhances our visual experience.

## 5. Conclusions

In this paper, research to improve the accuracy and flexibility of CGPSP has been conducted. A mathematical model was firstly derived to analyze the non-linearity error in the measured phases obtained by the CGPSP method. Then, the double five-step algorithm and the gamma correction method based on PDF were introduced to eliminate the non-linearity error. The double five-step algorithm proposed on the basis of the mathematical model can passively eliminate the second non-linearity error, while the gamma correction method can actively remove the high-order harmonics more effectively and requires fewer projected patterns. Therefore, CGPSP with the gamma correction method is recommended in practical applications. Finally, we applied the CGPSP method with gamma correction to the IPP technique, and the augmented reality display was realized. The experiment results demonstrate the CGPSP method with gamma correction is reliable and practical. The quantitative analysis of the geometric mapping accuracy between the projector pixel and camera pixel is not the main work of this manuscript. In the future work, we will research this problem.

## Figures and Tables

**Table 1:** Quantitative comparison of three methods when gamma value is 2.4.

Method	STD of the Horizontal Phase Errors (rad)	STD of the Vertical Phase Errors (rad)
Five-step CGPSP	19.557	19.557
Double five-step algorithm	0.046	0.046
Five-step SGPSP	0.002	0.002

**Table 2:** Pre-encoded values calculated from horizontal and vertical sinusoidal fringes.

Case	Pre-Encoded Values *γ_p_*	
	Horizontal Sinusoidal Fringe	Vertical Sinusoidal Fringe
1	1.701	1.698
2	1.697	1.689
3	1.680	1.673

**Table 3:** Quantitative comparison of measurement results by four methods.

Method	STD of the Horizontal Phase Errors (rad)	STD of the Vertical Phase Errors (rad)	Number of Fringe Patterns
Five-step CGPSP	12.974	11.711	15
Double five-step algorithm	0.035	0.037	30
Gamma correction	0.026	0.029	15
Five-step SGPSP	0.012	0.015	30

## Data Availability

Not applicable.
